# Efficacy and risk of mRNA vaccination in patients with autoimmune inflammatory rheumatic diseases

**DOI:** 10.1186/s41232-022-00247-1

**Published:** 2023-01-06

**Authors:** Yasuhiro Kato, Takayoshi Morita, Atsushi Kumanogoh

**Affiliations:** 1 Department of Respiratory Medicine and Clinical Immunology, Osaka University Graduate School of Medicine, Suita, Osaka, Japan; 2 Department of Immunopathology, Immunology Frontier Research Center (iFReC), Osaka University, Suita, Osaka, Japan; 3 Center for Infectious Diseases Education and Research (CiDER), Osaka University, Suita, Osaka, Japan; 4 Integrated Frontier Research for Medical Science Division, Institute for Open and Transdisciplinary Research Initiatives (OTRI), Osaka University, Suita, Osaka, Japan; 5 Japan Agency for Medical Research and Development — Core Research for Evolutional Science and Technology (AMED–CREST), Osaka University, Osaka, Japan; 6 Center for Advanced Modalities and DDS (CAMaD), Osaka University, Osaka, Japan

**Keywords:** COVID-19, SARS-CoV-2, Autoimmune inflammatory rheumatic disease (AIRD), mRNA vaccine, Immune response

## Abstract

Coronavirus disease 2019 (COVID-19), which spread worldwide from Wuhan, China, in 2019, appeared for a time to be overcome by the remarkable efficacy of mRNA vaccines; however, new variants of severe acute respiratory syndrome coronavirus 2 have emerged and remain rampant. The involvement of the virus in the emergence of variant strains and the relationship between vaccine efficacy and immunosuppressive drugs have attracted significant attention, particularly with regard to patients with autoimmune inflammatory rheumatic disease (AIRD) who take immunosuppressive drugs. This review outlines the relationship between mRNA vaccines, one of the key strategies against COVID-19, and AIRD and discusses the immune response elicited by mRNA vaccines. Furthermore, the impact of immunosuppressive agents on the mRNA vaccine-induced immune response in patients with AIRD and side effects of the vaccine, such as exacerbation of the underlying disease, is outlined.

## Background

The spread of severe acute respiratory syndrome coronavirus 2 (SARS-CoV-2) infection was considerably controlled owing to the remarkable efficacy of mRNA vaccines. However, new mutant strains, such as Delta and Omicron, have emerged and are spreading rampantly [1]. This may be attributed to viral factors, such as viral escape from vaccines encoding the wild-type S protein, and host factors, such as the gradual weakening of vaccine-induced immunity [2]. Patients with autoimmune inflammatory rheumatic disease (AIRD) treated with glucocorticoids or immunosuppressants are at high risk of severe coronavirus disease 2019 (COVID-19) and particularly need to be prevented from infection [3, 4].

In this review, we assess the latest research regarding the mRNA vaccine widely used to prevent COVID-19, the effect of immunosuppressive agents used to treat AIRD on vaccine efficacy, and the adverse effects of vaccination, including exacerbation of the underlying disease owing to the immunostimulatory effect of the vaccines.

## AIRDs and their relationship to COVID-19

**Fig. 1:**
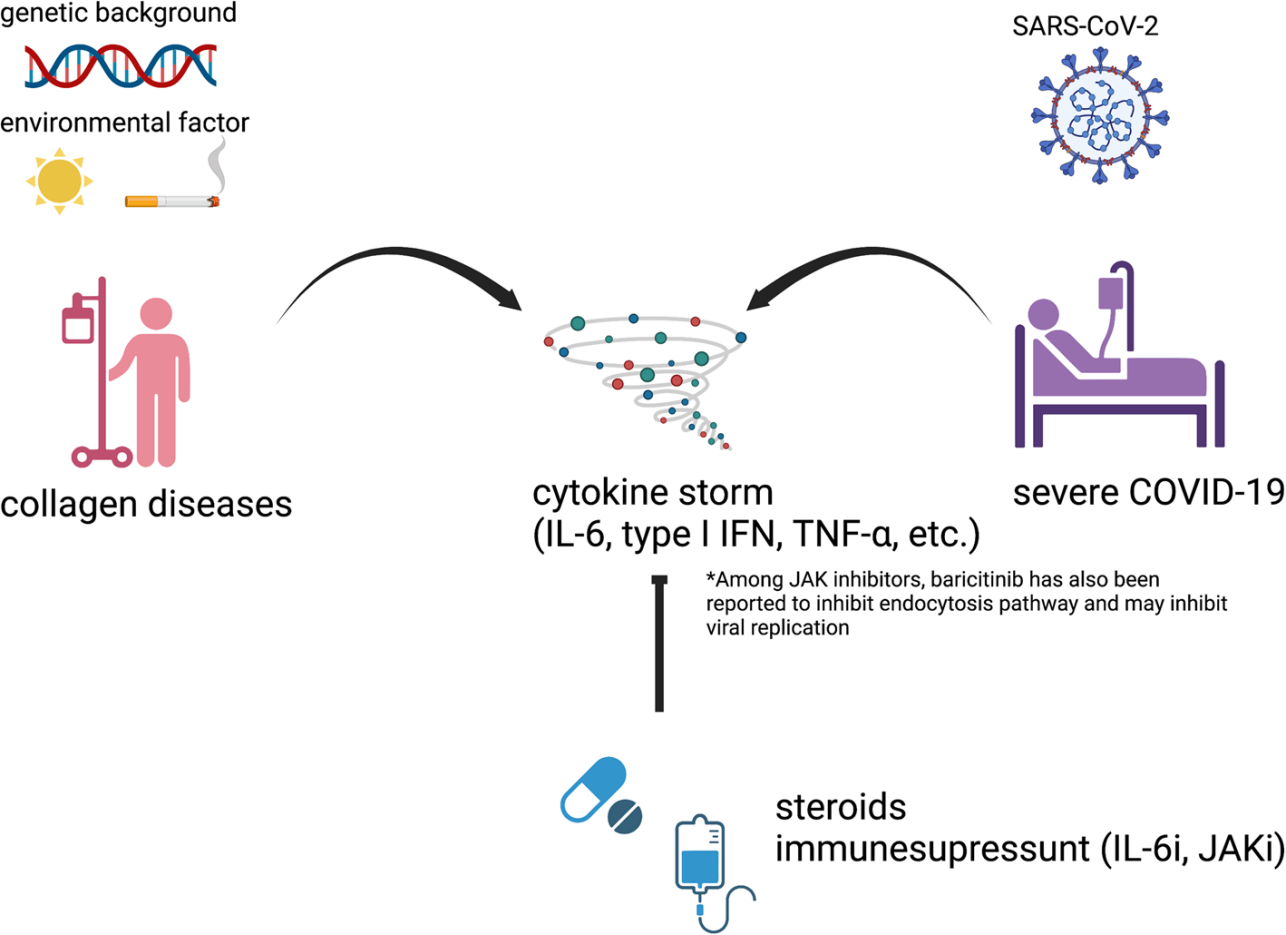
Commonalities between AIRD and COVID-19. Both AIRDs, which develop from genetic background and environmental factors, and COVID-19, which results from SARS-CoV-2 infection, are known to cause cytokine storms. In common, they are treated with glucocorticoid, JAKi, or IL-6. Among JAKis, baricitinib has also been reported to inhibit endocytosis pathway and may inhibit viral replication. COVID-19 coronavirus disease, SARS-CoV-2 severe acute respiratory syndrome coronavirus 2, IL-6 interleukin-6, IFN interferon, TNF tumor necrosis factor, JAK Janus kinase

AIRDs are multi-organ disorders caused by an abnormal immune response to self-antigens. The pathophysiology of AIRDs is closely related to that of COVID-19. Intense immune responses to SARS-CoV-2 induce cytokine storm [5, 6]. Likewise, autoimmune diseases are also complicated by cytokine storms, known as macrophage activation syndrome (MAS) [7]. Furthermore, the biological functions of cytokines such as interleukin-6 (IL-6), type 1 interferon (IFN), and tumor necrosis factor-alpha (TNF-α), which constitute the cytokine storm, have been elucidated by research and clinical practice in autoimmune diseases [8]. Therapies used to treat severe COVID-19, including glucocorticoids, IL-6 inhibitor, Janus kinase inhibitors (JAKi), and plasma exchange, are also widely used to treat AIRDs [9] (Fig. 1). Among JAKis, baricitinib has also been reported to inhibit AP2-associated protein kinase 1 (AAK1) and cyclin G-associated kinase (GAK), potentially inhibiting viral replication [10]. Interestingly, reports on COVID-19 susceptibility also indicate that SLE genetic predisposition may be protective against COVID-19 [11].

Furthermore, persistent infection in immunocompromised patients has been reported to be one of the causes of the emergence of variants of concern. Choi et al. reported viral mutation within an individual owing to the persistence of infection without viral elimination [12]. Notably, mutations resulting from persistent infection in immunocompromised patients confer resistance to neutralizing and clinically administered monoclonal antibodies against SARS-CoV-2 isolated from convalescent COVID-19 patients [13]. In one patient with hypogammaglobulinemia following B-cell removal therapy, SARS-CoV-2 infection persisted for 101 days despite convalescent plasma and remdesivir administration. In this patient, D796H in the S2 subunit and a deletion (ΔH69/ΔV70) in the S1N-terminal domain of the spike protein reportedly conferred resistance to the neutralizing activity of convalescent plasma/serum from several patients while maintaining the same infectivity as the wild type in vitro [14].

Thus, our knowledge of the immune response in AIRD may provide insight not only into the pathogenesis of COVID-19 but also into the mechanisms of appearance of clinically and socially important mutant strains. However, the pathophysiology of AIRD itself remains to be fully elucidated and awaits further research.

## Immune responses to the mRNA vaccine

mRNA vaccines, such as BNT162b2 (Pfizer, BioNTech), consist of lipid nanoparticles (LNPs) and mRNA encoding the SARS-CoV-2 spike protein [15]. The mRNA is translated into the spike protein, thereby providing specific antigen stimulation. The mRNA may also act as an adjuvant by stimulating RNA sensors such as toll-like receptor (TLR)-3, TLR-7, and malondialdehyde (MDA)-5 [16]. LNPs also reportedly have an adjuvant effect that enhances the efficacy of mRNA vaccines [17].

In an analysis of the immune response following BNT162b2 vaccination in healthy post-vaccine volunteers, Arunachalam et al. reported that vaccination results in an enhanced adaptive immune response via the production of neutralizing antibodies against wild-type SARS-CoV-2 and an increase in the number of antigen-specific CD4 and CD8 T cells after the second dose [18]. Furthermore, the response of the innate immune system is enhanced after the second dose of mRNA vaccine, as observed by elevated IFN-γ in the blood and increased inflammatory monocytes, characterized by IFN-related gene expression [18]. Bacille Calmette-Guérin vaccines have been reported to enhance the response of the innate immune system following vaccination, termed “trained immunity,” and have been reported to cause epigenomic changes in monocytes [19, 20]. mRNA vaccines may also influence the memory of innate immunity as well as acquired immunity. Further research is warranted to elucidate the mechanisms underlying immune responses to mRNA vaccines.

## Effect of immunosuppressant use on vaccine efficacy

The widespread use of vaccines is a crucial strategy to control SARS-CoV-2 infection. To evaluate the effectiveness of vaccines, infection prevention rates need to be assessed; however, large-scale trials are required [21, 22]. Therefore, directly evaluating the preventive effects of vaccines in patients with AIRDs is challenging owing to the limited number of such patients. However, neutralizing antibody titers induced by vaccination can be readily evaluated and are thought to correlate with the efficacy of infection prevention [23]. Therefore, most studies of vaccine efficacy in patients with AIRDs assess antibody titers, such as those of neutralizing antibodies.

A study of mRNA and viral vector vaccines assessed the antibody titer of serum IgG against the SARS-CoV-2 receptor-binding domain (RBD) in 90 patients with systemic lupus erythematosus (SLE). The antibody titer was below the lower limit of healthy controls in 28.8% of SLE patients. Antibody titers of IgG against the RBD correlated with the capacity of T-cell IFN-γ production as measured by enzyme-linked immunospot (ELISpot) assays. Both antibody titers and IFN-γ production capacity were reduced in some SLE patients [24]. In another study involving 126 SLE patients, the use of mycophenolate mofetil (MMF) and methotrexate (MTX) was associated with decreased serum neutralizing activity, and baseline naive B-cell frequency was correlated with neutralizing activity [25].

In a study assessing the titer of serum IgG antibody against the SARS-CoV-2 spike protein in 686 patients with AIRD after vaccination, the seropositivity rate and IgG antibody titer were significantly lower than those in the control group. Factors contributing to reduced immunogenicity included advanced age and treatment with glucocorticoids, rituximab, MMF, and abatacept. Notably, rituximab treatment was the leading cause of negative seroreactivity (39% seropositivity) [26]. In a study evaluating the response to mRNA or viral vector vaccines in 140 participants receiving immunosuppression treatment for autoimmune rheumatic and glomerular diseases, the antibody positivity rate was 59.3%, and the T-cell response rate was 82.6%. Notably, seroconversion did not occur when the level of B-cells was reduced at the time of vaccination, and the T-cell response was reduced when tacrolimus was used, indicating that different types of immunosuppressive agents can cause differences in humoral and cellular immune responses. In this study, in 8.7% of patients, neither antibody nor T-cell responses were detected after the second vaccination [27]. In a systematic review of patients with immune-mediated inflammatory diseases, the seroconversion rate in the group of patients treated with anti-CD20 (rituximab) or anti-cytotoxic T-lymphocyte associated antigen (abatacept) therapies was less than 70%, which is reduced compared with other treatment groups [28].

Collectively, two doses of mRNA vaccines may be insufficient to achieve a vaccine effect in some patients. However, a case report demonstrated a sufficient increase in the neutralizing antibody titer after four vaccinations [29]. Therefore, investigating the possibility of achieving a booster effect via multiple vaccinations is crucial. As discussed in a later section, the European Alliance of Associations for Rheumatology (EULAR) recommends three doses of mRNA vaccines for patients receiving immunosuppressive drugs.

## Vaccine side effects in patients with AIRDs

Immune responses that occur after vaccination reportedly cause increases in the levels of serum inflammatory cytokines, such as IFN-γ, and activation of the innate immune system [18]. In contrast, no apparent increase in the serum level of type 1 IFN, which is crucial in the pathophysiology of AIRDs such as SLE, was observed after vaccination, possibly owing to insufficient measurement sensitivity [18]. The vaccine-induced inflammation is usually self-limiting; however, in some cases, the persistence of inflammation leads to the exacerbation or development of rheumatic disease.

In an observational study of 90 SLE patients (among which 79 were evaluated for post-vaccine adverse effects), disease exacerbations following mRNA or viral vector vaccination were reported in 11.4% of patients; 1.3% of these cases were severe. However, this 1.3% comprised an arthritis case rather than an organ damage case [24]. In a study of 126 SLE patients, there was no apparent British Isles Lupus Assessment Group (BILAG) or Systemic Lupus Erythematosus Disease Activity Index (SLEDAI) exacerbation at the 2-week follow-up after the second vaccination [25]. Approximately 20% of patients with rheumatoid arthritis exhibited worsening of the disease after vaccination; however, the rate of exacerbation was lower in patients with psoriasis, ankylosing spondylitis, or SLE than in patients with rheumatoid arthritis, suggesting that the disease activity in AIRDs is generally stable after mRNA vaccination [26]. A survey of 1377 patients with rheumatic and musculoskeletal diseases (RMD) suggested that the rate of treatment-requiring relapse after mRNA vaccine administration was 11%, with no reports of severe relapse [30].

Based on these reports, the risk of vaccine-induced exacerbation of the underlying disease is considered to be lower than the risk of severe COVID-19, and vaccination is recommended. However, there have been some reports of AIRD-like symptoms occurring after vaccination, even in patients without AIRD [31–34], as well as cases of highly inflammatory polyarthritis reminiscent of polymyalgia rheumatica and worsening of interstitial pneumonia that we reported in a previous study [35, 36].

## Current opinions for vaccination of patients with AIRDs

Patients with AIRDs exhibit reduced vaccine efficacy with the potential for side effects. The current opinions for administering vaccines to patients with rheumatic diseases are described as follows:

The EULAR notes that, as of February 2022, there are insufficient data on the COVID-19 vaccine status in patients with rheumatic diseases and those receiving immunosuppressive treatment; however, several vaccines can be safely administered as we lack evidence to discourage their use, especially in patients with RMD. Precautions include administering the vaccine when the disease is stable or before the start of treatment if the patient is receiving immunosuppressive drugs intermittently. The organization also suggests that immunosuppressive drugs other than rituximab should not be discontinued to facilitate vaccination. Furthermore, a single vaccination schedule, which includes a third dose at least one month after the second dose, is recommended to prevent weakening of vaccine efficacy owing to long-term use of rituximab, cyclophosphamide, MMF, abatacept, or prednisone at a dose of ≥ 10 mg/day in most countries.

The American College of Rheumatology (ACR) published the COVID-19 vaccine clinical guidance summary for patients with rheumatic and musculoskeletal diseases, version 5, in February 2022. This publication states that the benefits of COVID-19 vaccination outweigh the associated risk of developing new autoimmune diseases, thereby encouraging the administration of vaccines (level of task force consensus: moderate). The report also mentions that some immunosuppressive drugs should be paused, assuming the disease is well-controlled (level of task force consensus: moderate).

Both the EULAR and the ACR have taken positions recommending vaccination for patients with AIRD, and clinicians should consider these comments when deciding whether to vaccinate a patient.

## Conclusions

The mRNA vaccine is highly effective; however, its efficacy is attenuated in patients undergoing immunosuppressive therapy. In addition, the long-term effects are not well known; thus, the vaccine should be administered with caution. We have analyzed the long-term effects of neutralizing antibody titers in vaccinated patients with AIRD and observed that levels of neutralizing antibody titers decrease earlier in patients with AIRD compared with healthy volunteers, and that there is no early weakening of antibody titers in TNFi, rather a long-term decreasing trend (unpublished data). In addition, most of the previous reports on immune responses to vaccines in patients with AIRD have been related to the acquired immune system, such as humoral immunity, but there are few reports on innate immune memory. Innate immune memory, also called “trained immunity,” is an immunological memory in which changes in innate immune cell function occur after infection or vaccination, increasing responsiveness to restimulation, similar to the adaptive immune memory of B cells and T cells. With regard to the memory of the innate immune system by the vaccination, we have found that the chromatin of the type 1 IFN signature gene in the monocyte lineage opens after the vaccination of the healthy volunteers, and that this change is transient [37]. These findings may provide information regarding the optimal administration of vaccines and encourage continued studies of mRNA vaccines in patients receiving immunosuppressive treatment for AIRDs. However, we lack sufficient data on the efficacy and safety of the COVID-19 vaccine in these patients, and strategies for maximizing vaccine efficacy and achieving long-term efficacy have yet to be developed. The accumulation of further data would facilitate the recommendation of suitable vaccines and vaccination intervals depending on the underlying disease and associated treatment for patients with AIRDs.

## Data Availability

Not applicable.

## Abbreviations

AAK1: AP2-associated protein kinase 1
ACR: American College of Rheumatology
AIRD: Autoimmune inflammatory rheumatic disease
BILAG: British Isles Lupus Assessment Group
COVID-19: Coronavirus disease 2019
CTLA-4: Cytotoxic T-lymphocyte-associated antigen
EULAR: European Alliance of Associations for Rheumatology
GAK: Cyclin G-associated kinase
IFN: Interferon
JAKi: Janus kinase inhibitors
LNPs: Lipid nanoparticles
MDA: Malondialdehyde
MMF: Mycophenolate mofetil
MTX: Methotrexate
RBD: Receptor-binding domain
RMD: Rheumatic and musculoskeletal diseases
SARS-CoV-2: Severe acute respiratory syndrome coronavirus 2
SLE: Systemic lupus erythematosus
SLEDAI: Systemic Lupus Erythematosus Disease Activity Index
TCZ: Tocilizumab
TNF: Tumor necrosis factor
